# Mass spectrometry detects folding intermediates populated during urea-induced protein denaturation

**DOI:** 10.1039/d5sc05773f

**Published:** 2025-10-23

**Authors:** Nicklas Österlund, Jacob S. Jordan, Eleonora Renzi, Gergo Peter Szekeres, Kevin Pagel

**Affiliations:** a Institute of Chemistry and Biochemistry, Freie Universität Berlin Berlin Germany kevin.pagel@fu-berlin.de; b Department of Molecular Physics, Fritz Haber Institute of the Max Planck Society Berlin Germany

## Abstract

Protein folding stability can be probed using urea, a chaotropic agent that disrupts non-covalent interactions at molar concentrations. The denaturation process is typically monitored *via* optical spectroscopy, which provides ensemble-averaged measurements and may struggle to resolve folding intermediates. In contrast, electrospray ionization mass spectrometry (ESI-MS) captures a non-averaged snapshot of all populated assembly and folding states within a protein conformational ensemble. However, high urea concentrations have traditionally been considered incompatible with ESI. Here, we leverage recent advancements in nano ESI emitter design, utilizing well-defined small-diameter emitters which enables protein charge states to be resolved from solutions containing up to 8 M urea. This approach allows us to directly detect the disruption of native tertiary and quaternary structures and to monitor stability changes in response to solution pH and ligand binding. We demonstrate this using single-domain proteins that follow simple two-state unfolding pathways, as well as more complex multidomain proteins and multimeric protein complexes. Our results show strong agreement with conventional urea–denaturation curves obtained *via* optical spectroscopy, while also providing enhanced resolution of intermediate folding and assembly states that are challenging to capture using traditional methods.

## Abstract 2

Nano ESI enables direct detection of protein folding and assembly states in solutions containing up to 8 M urea, revealing structural intermediates and stability changes inaccessible to traditional spectroscopy approaches.

## Graphical abstract

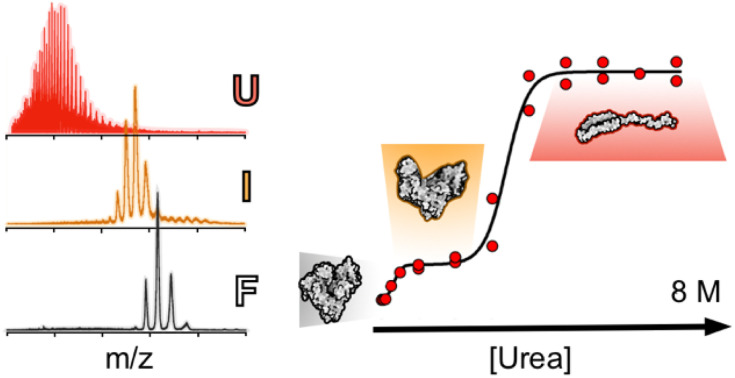

## Introduction

Proteins fold into specific three-dimensional structures that are intricately linked to their native biological functions. These structures are typically marginally stable under physiological conditions and can be readily perturbed,^1^ leading to protein misfolding and aggregation associated with the development of proteinopathies.^2,3^

To probe stability *in vitro*, the equilibrium between folded and unfolded states can be shifted using chaotropic agents such as urea, which disrupt native non-covalent interactions in a concentration-dependent manner.^4,5^ Titration with urea is one of the most widely used experimental strategies in biochemistry, providing tuneable perturbations that yield quantitative parameters such as unfolding free energies and cooperativity.^6^ These measurements are routinely applied to understand how, for example, mutations, ligand binding, or posttranslational modifications influence the energetic balance of folding.^7–10^ Importantly, direct *in vivo*/*in vitro* comparisons have demonstrated that such denaturant-derived *in vitro* stabilities typically correlate well with protein stability behaviour in cells.^11–13^

Protein unfolding is routinely investigated using optical spectroscopy techniques, including fluorescence or circular dichroism (CD) spectroscopy, which monitor solvation-dependent changes in protein fluorescence and protein secondary structure, respectively.^14,15^ While these methods generally provide high quality data, it is important to note that they measure ensemble-averaged properties and struggle to resolve low-abundance species, such as transient folding intermediates, or heterogeneous populations that arise during unfolding. Such low-populated intermediates are of particular interest in the disease mechanisms for several proteinopathies due to their high toxicity.^16–18^

Native mass spectrometry (MS) is a powerful technique with the capacity to detect low abundance species, and the ability to resolve different protein complex assembly states as long as they differ in their mass-to-charge (*m*/*z*) ratio.^19,20^ Native MS has enabled the characterization of the stoichiometry, shape, gas-phase stability, and ligand-binding affinity of numerous proteins, as well as protein–protein and –RNA/DNA complexes.^21^ Using electrospray ionization (ESI), native-like protein structures can be retained upon ionization and transfer to the gas phase.^22–24^ In combination with ion mobility spectrometry (IMS), which reports on the rotationally averaged shape of ions in the gas phase, the conformational ensembles of a number of model proteins has been investigated in detail.^25–27^

Modifications to the electrospray process can be used in combination with IMS-MS measurements to determine the thermal or chemical stability of proteins in solution. Protein ions produced from solutions at low or high pH,^28,29^ or at high temperatures,^30^ have higher charges than those produced from neutral pH or at room temperature. These experiments indicate that the ESI charge state distribution (CSD) is a reporter on the surface area, and hence folding state, of the protein.^31–33^ Importantly, because ionization occurs during the final stages of solvent evaporation, the CSD can serve as a proxy for the protein's solution-state conformation at the moment of ionization, provided that the droplet composition does not contain other species capable of altering the protein's charging behavior.^34–38^

Despite its potential, the application of ESI-MS to monitor urea-induced denaturation has been limited due to the high osmolyte concentrations required for protein unfolding.^39,40^ Traditionally, high-millimolar to molar concentrations of additives have been considered incompatible with ESI, as these conditions can suppress protein ionization and compromise spectral quality by introducing chemical noise. These challenges primarily arise from the excessive presence of additives in the electrospray-generated nanodroplets, where they either compete with analytes for ionization or form nonspecific clusters that obscure protein signals in the resulting mass spectra.^41–44^

The use of small diameter nano-ESI (nESI) emitters has proven effective in minimizing such matrix effects that compromise mass spectral quality. By reducing the emitter size, conditions can be obtained such that ESI droplets contain, on average, fewer than one analyte of interest.^45,46^ The droplets that do contain a protein analyte also contain fewer interfering additives. This enables protein mass spectra to be obtained even from solutions containing >150 mM of nonvolatile salts and buffers.^45,47^

In this study, we leverage these recent advancements in nESI emitter design^45,47–49^ to acquire protein mass spectra with resolved charge state distributions directly from solutions containing up to 8 M urea. Using this approach, we directly observe the stepwise loss of native tertiary and quaternary structures with enhanced resolution of intermediate folding states. We demonstrate this for simple single domain proteins which undergo two-state unfolding pathways, as well as for multidomain proteins that display more complex unfolding behavior and multimeric protein complexes where the stabilities of individual assembly states can be tracked independently. Comparison with conventional optical spectroscopy measurements confirms that nESI-MS provides a complementary and highly sensitive method for investigating protein unfolding and stability.

## Results and discussion

### Probing proteins by nESI from molar concentrations of urea

The ability of nESI-MS to capture urea-induced protein unfolding was explored using the small globular protein myoglobin. Myoglobin (Mb) is an excellent model protein in native MS, as it contains a non-covalently bound heme-group which dissociates upon denaturation. Monitoring the relative intensities of Mb containing (holo, hMb) and missing (apo, aMb) the heme group in the mass spectrum can thus be used as a sensitive reporter for denaturation.^24,50,51^

**Fig. 1:**
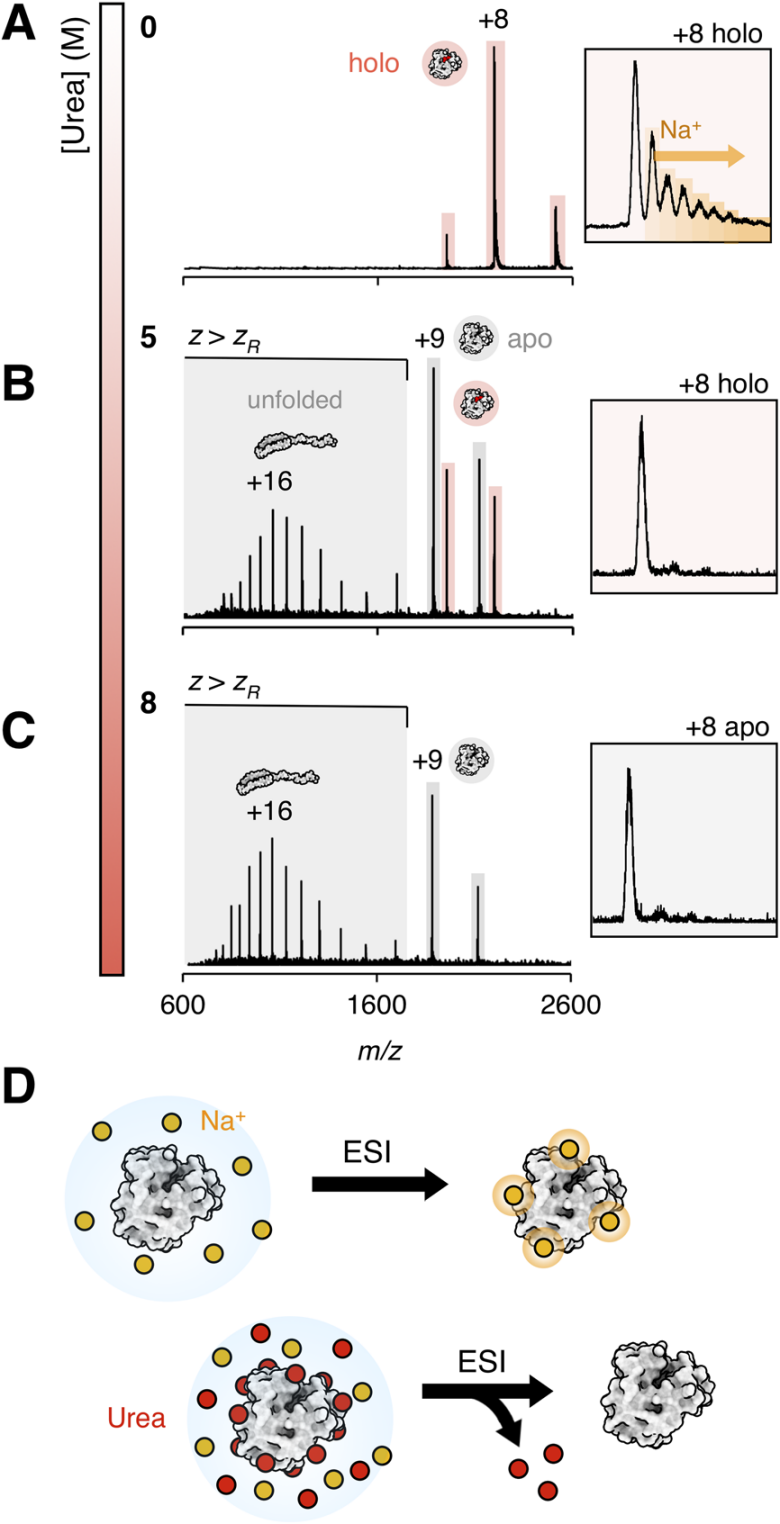
nESI-MS of myoglobin (Mb) in 200 mM ammonium acetate (pH 6.8) with (A) 0 M (B) 5 M and (C) 8 M urea. Inserts show the +8 charge state. Heme-bound myoglobin (hMb) is highlighted in red, heme-free (aMb) myoglobin is highlighted in grey. Signals where the ESI charge (*z*) is higher than the theoretical Rayleigh charge (*z*_R_) for myoglobin (+9.8) are indicated. (D) Proposed mechanism for the desalting effect observed in the presence of urea.

Mb was analyzed on a Synapt G2-S IMS-MS instrument (Waters Corp.), by spraying the protein in 200 mM ammonium acetate (pH 6.8) from nESI emitters with an inner diameter of approximately 1 μm. Instrument conditions were tuned to minimize gas-phase protein unfolding and heme dissociation. This yielded a narrow charge state distribution of predominantly hMb, centered around the +8 charge state (Fig. 1A), which is in agreement with prior native mass spectra acquired on this protein.^32^

Addition of increasing amounts of urea resulted in a clear shift towards the apo form, as well as a shift towards higher charge states (Fig. 1B and C). Such shifts towards higher ESI charge states are indicative of a loss of compact tertiary structure in solution as ESI charge correlates with protein solvent accessible surface area in the electrospray droplet.^52^ In general, charge states exceeding the Rayleigh charge (*z*_R_) of the protein, such as those observed for Mb between 600 and 1600 *m*/*z* at 8 M urea, are formed from extended and unfolded conformations.

By contrast, the ion mobility arrival time distributions for Mb change only marginally with increasing urea concentration, both for aMb and hMb and across charge states above and below *z*_R_ (Fig. S1). This indicates that each ESI charge state corresponds to a specific solution-state conformation, and that denaturation mainly alters the relative abundance of these solution-state conformations within the ensemble, rather than their corresponding gas-phase conformation as seen by IMS. This shift within the conformational ensemble is in turn detected as a shift in the average charge-state of the charge-state distribution (CSD) in the mass spectrum. The increased abundance of signals above *z*_R_ with higher urea concentrations shows that the products of urea-induced unfolding can be readily probed by nESI and followed directly from the MS data.

Remarkably, the spectral quality remained excellent even in 8 M urea, with high spray stability and low signal variability both within and among replicates. This effect was achieved on both the Synapt platform and a Q Exactive UHMR MS instrument (Thermo Scientific) equipped with an orbitrap mass analyser (Fig. S2). On the Q Exactive, where the ESI needle is positioned on-axis with the MS inlet, a slight left–right needle offset in respect to the inlet could sometimes improve spectral quality in the presence of urea. Such needle positioning more closely resembles the geometry of the Waters ESI source used on the Synapt platform, where the needle is oriented at 90° relative to the inlet, and likely reduces the amount of urea entering the mass spectrometer.

A clear decrease in sodium adduction was even observed upon urea addition (Fig. 1A–C, inserts). At 0 M urea, approximately 60% of the ion population consisted of sodium-adducted species, whereas at urea concentrations of 1 M and above, nearly all detected protein ions appeared salt-free. This likely occurs as urea outcompetes the salts at the protein surface prior to final desolvation from the electrospray droplet (Fig. 1D). We tested this by measuring the mass spectrum of Mb at physiological NaCl concentrations (150 mM) and in more biochemically relevant sodium phosphate buffers (20 mM, pH 7.5). No mass spectrum could be measured without urea, while well resolved, although sodium adducted, Mb peaks could be detected after addition of 1 M urea (Fig. S3), showing that urea does have a clear desalting effect in ESI-MS of proteins. This effect is similar to “buffer loading” strategies used to mitigate metal ion-induced signal suppression in ESI.^53^ However, urea offers the added advantage of stronger interactions with the protein surface, enabling effective desalting and spectral acquisition even in the presence of higher concentrations of non-volatile salts.

Difficulties in ionizing proteins at urea concentrations above 3 M have previously been reported.^39^ In those experiments, nESI emitters were fabricated with closed tips that were manually clipped open under a light microscope. Microscopy imaging of emitters prepared in this manner revealed an inner diameter of 12.3 μm (Fig. S4A), approximately ten times larger than that of the emitters used in our experiments (1.2 μm) (Fig. S4B). These larger emitters also failed to produce well-resolved mass spectra of Mb in 5 M urea (Fig. S4C and D). This indicates that well-defined, small-diameter nESI emitters are essential for obtaining high-quality mass spectra of proteins in the presence of molar concentrations of urea.

### Monitoring in-solution unfolding stability in the gas phase

**Fig. 2:**
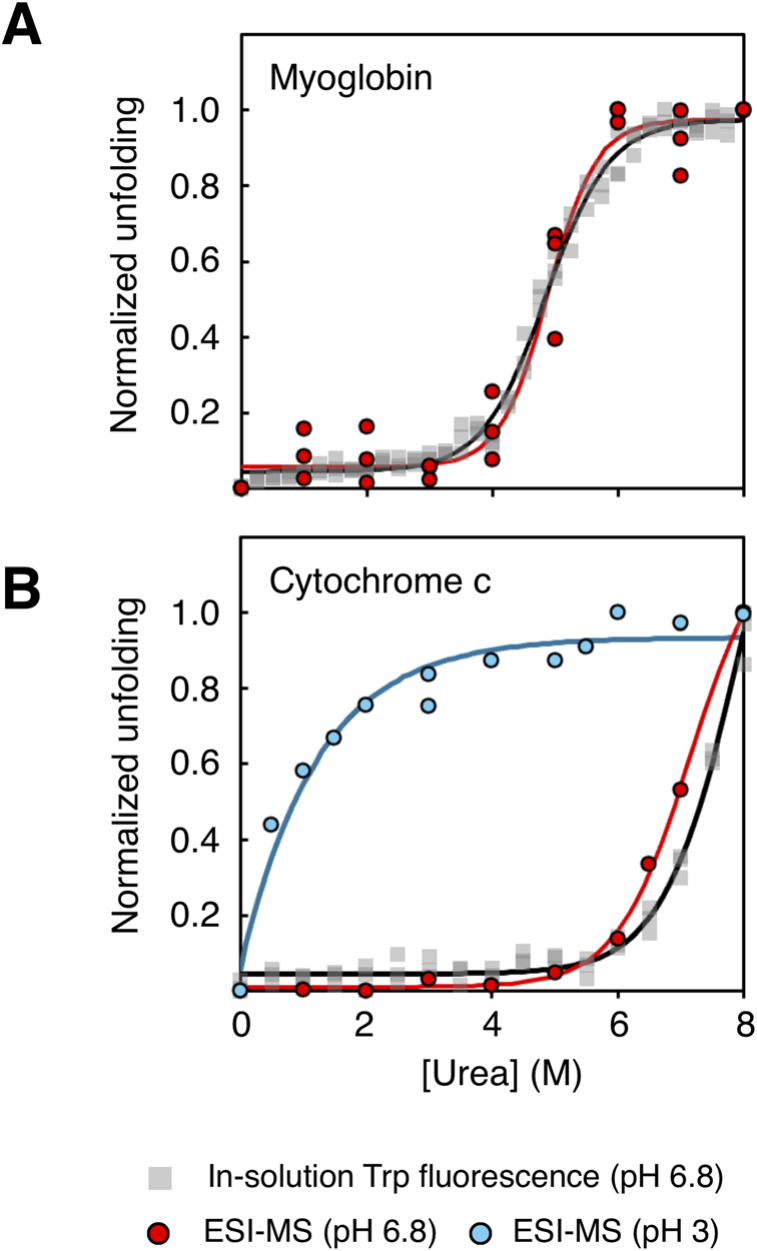
Normalized stability curves from ESI-MS experiments (circles) based on changes in the average charge state or protein ions, and in solution fluorescence (squares) monitoring the 354/338 nm emission ratio after excitation at 280 nm. The data were normalized to enable visual comparison between ESI-MS and fluorescence data. Normalization was performed such that, within each replicate, the lowest y value was set to 0 and the highest value to 1. (A) Stability curves for myoglobin in 200 mM ammonium acetate pH 6.8, measured in solution (black) and in the gas-phase by monitoring the apo/holo ratio of the protein (red). (B) Stability curves for cytochrome c in 200 mM ammonium acetate, measured in solution (black) and in the gas-phase by monitoring the average charge state of the protein at pH 6.8 (red) and at pH 3 (blue).

As mass spectra could be obtained over a broad range of urea concentrations, this allowed us to construct an unfolding curve for Mb based on the population abundance of the apo form (Fig. 2A, red). Fitting of the data to a two-state model yielded a midpoint of unfolding at 4.9 M urea. The fitted parameters were highly reproducible across three independent replicates, each measured on separately prepared samples on different days (Fig. S5), with a coefficient of variation of 5.1% for the unfolding midpoint. The obtained midpoint is also in excellent agreement with data obtained in solution under identical sample conditions, by monitoring tryptophan fluorescence, also yielding an unfolding midpoint at 4.9 M urea (Fig. 2A, black).

The average ESI charge of Mb increased from +8.5 (0.87 *z*_R_) at 0 M urea to +13.8 (1.4 *z*_R_) at 8 M. A sigmoidal fit of the total charge state distribution (CSD) for Mb at different urea concentrations reproduces the curve obtained by following heme dissociation (Fig. S6A), indicating that high concentrations of urea do not affect the charging during ESI. Furthermore, monitoring the CSD enabled us to resolve the individual stabilities of the apo and holo forms of Mb. We found that aMb displays a sharp unfolding transition at 4 M urea, which is the onset of heme dissociation, while the holo form remains in a compact conformation at all urea concentrations where it is observed (Fig. S6B). This illustrates that the loss of heme binding greatly destabilizes Mb.

It should however be noted that ∼30% of aMb remains as low charged states, with an ESI charge lower than the theoretical *z*_R_ for myoglobin (+9.8), upon reaching the post-transition phase of the unfolding curve (Fig. 1C and S7A–B). This indicates that the folding equilibrium at 8 M urea is not fully shifted towards the globally unfolded state, as is observed in the mass spectrum of acid denatured Mb (Fig. S7C). Such direct information on the conformational ensemble is not obtainable by typical spectroscopic methods, where the post-transition phase is often interpreted as the fully unfolded state.^15^ Similar levels of compact species have been observed in the gas phase for model proteins at elevated temperatures, suggesting that native-like structures may be more stable than standard ensemble-averaging methods imply.^54^

As we observe that the ESI charging mechanism is not affected by the presence of urea (*i.e.* charge reduction or supercharging), this MS-based approach should be broadly applicable also to proteins lacking non-covalently bound cofactors. Therefore, we next tested the method on cytochrome *c* (Cyt *c*), a small globular protein with a covalently bound heme group. Compared to Mb, Cyt *c* exhibited significantly greater stability, with the onset of unfolding detected only at urea concentrations above 6 M, as determined by both mass spectrometry (Fig. 2B, red) and fluorescence spectroscopy (Fig. 2B, black).

Variable temperature ESI experiments have shown that Cyt *c* can be greatly destabilized by a drop in pH.^54^ When acidifying Cyt *c* to pH 3, the CSD shifts to higher charge states while still populating compact states close to *z*_R_ (Fig. S8A). This is in agreement with a molten globule state, which has also been previously reported for Cyt *c* at low pH.^55^ Addition of increasing amounts of urea shows that this molten globule form displays significantly lower folding stability compared to the native state populated at neutral pH (Fig. 2B, blue). The lower pH also induces formation of dimeric and trimeric states (Fig. S8A), which has been attributed to domain-swapping under these non-native conditions.^56^ Mass spectrometric detection here allows us to simultaneously follow the unfolding of these misfolded monomers and dimers (Fig. S8B), which is not typically possible using solution-phase ensemble-averaging techniques.

### Detection of a partially folded intermediate by MS

While small single domain proteins, such as Mb and Cyt *c*, often unfold by a simple two-state mechanism, other proteins are known to populate intermediate states during unfolding.^57,58^ This is especially common for larger multidomain proteins.^59,60^ As ESI-MS reports on the entire protein ensemble and not on its average properties, such intermediates should be observable even if they only populate a small percentage of the protein ensemble, or if they represent a minor shift in protein conformation.^61^ This was explored using bovine serum albumin (BSA), which consists of three domains, by acquiring mass spectra in 200 mM ammonium acetate (pH 6.8) and urea concentrations between 0 and 8 M.

**Fig. 3:**
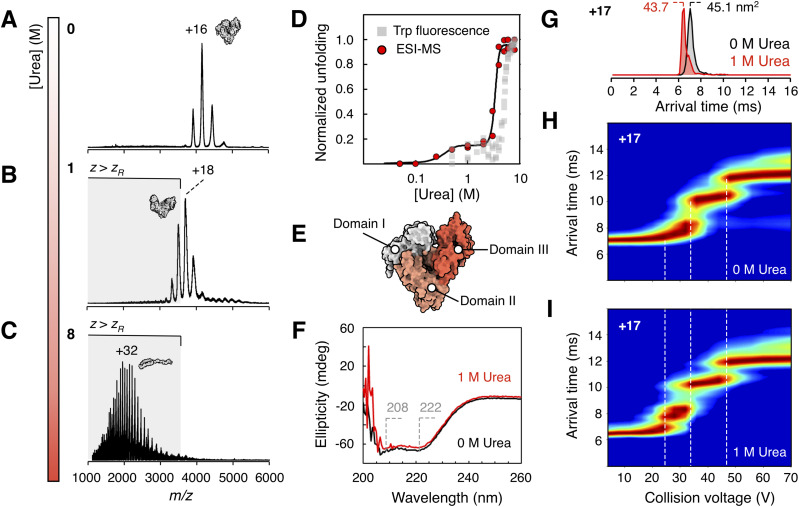
nESI-MS of BSA in 200 mM ammonium acetate (pH 6.8) with (A) 0 M (B) 1 M and (C) 8 M urea. (D) Normalized stability curves from monitoring the average charge state in gas-phase experiments (circles), and in solution fluorescence (squares) monitoring the 354/338 nm emission ratio after excitation at 280 nm. The data were normalized to enable visual comparison between ESI-MS and fluorescence data. Normalization was performed such that, within each replicate, the lowest *y* value was set to 0 and the highest value to 1. The gas-phase data is fitted to a two-component sigmoidal function (black line). (E) Structure of BSA (pdb 3v03)^79^ with its three domains indicated. (F) Circular dichroism spectrum of BSA in 200 mM ammonium acetate (pH 6.8) with 0 M (black) and 1 M (red) urea. The minima at 208 and 222 nm, characteristic for a helical structure, are marked. (G) Ion mobility arrival time distribution of the +17 BSA charge state with 0 M (black) and 1 M (red) urea. Shorter arrival times correspond to a smaller collision cross section. CIU plots of the +17 BSA charge state in (H) 0 M (I) 1 M urea. Gas-phase unfolding is monitored as a function of applied collision voltage in the trap compartment of the mass spectrometer.

Mass spectra of BSA acquired from 0 M urea indicate a fully folded monomer ensemble with a narrow CSD with an average charge of +15.7 (0.82 *z*_R_) (Fig. 3A). Increasing the urea concentration led to a gradual shift of the CSD towards higher charge states. Low urea concentrations widen the CSD slightly and shift it to an average charge of +18.5 (0.97 *z*_R_) (Fig. 3B), similar to the molten state observed for cytochrome *c* at low pH. Increasing the urea concentration further leads to a wide and highly charged distribution, with an average charge state of +32 (1.7 *z*_R_), indicative of a globally unfolded state (Fig. 3C).

Each urea concentration was measured in independent duplicates, yielding highly consistent charge state distributions. Fitting of the average charge states of BSA to a two-component sigmoidal model shows that the first unfolding transition occurs at a transition midpoint of 0.33 M urea. This state is stable until the second unfolding event with a transition midpoint of 3.4 M (Fig. 3D, red). The presence of a stable plateau phase between 0.5 and 2 M urea is directly indicative of an unfolding intermediate. This intermediate structure cannot be clearly detected by solution-state fluorescence, which shows a distinct increase only during the second unfolding transition (Fig. 3D, grey). However, subtle baseline shifts at lower urea concentrations may indicate conformational changes.

This unfolding intermediate may arise due to partial unfolding of one of the domains in BSA. The three domains (I–III) (Fig. 3E) are structurally homologous but differ in stability, with domain III being the least stable.^62^ Structural rearrangements in domain III have been observed at low osmolyte concentrations, and a hydrophobic binding site between the domains becomes accessible under mild denaturation conditions.^63^ Therefore, it is likely that the minor transition observed by nESI-MS at 0.33 M urea corresponds to a slight expansion of the space between domains.

Circular dichroism (CD) spectroscopy reveals no significant change in secondary structure at low urea concentrations, as BSA retains a predominantly α-helical conformation with characteristic minima at 208 and 222 nm (Fig. 3F). Instead, CD only reports a major transition corresponding to the second unfolding event (Fig. S9). These findings suggest that the protein remains largely folded at the domain level during the first transition, while possibly losing tertiary interdomain contacts. In contrast, the second transition reflects a more extensive unfolding process in which intradomain structures are disrupted, leading to a fully unstructured and extended conformation.

To probe the structure and stability of the intermediate state we next employed ion mobility spectrometry (IMS). For the +17 charge state, a slight compaction of 144 Å^2^ could be observed in the arrival time distribution, yielding a collision cross-section (CCS) that corresponds to 97% of the value measured for the native-like ion in the absence of urea (Fig. 3G). Such gas-phase compaction is known to occur for unstructured protein regions,^64^ and could here be due to the dehydration and partial collapse of a swollen structure with disrupted interdomain contacts. Collisional activation within the mass spectrometer leads to a slow heating of the protein, inducing unfolding events that are detectable by IMS.^65^ By monitoring changes in ion mobility as a function of stepwise increases in activation energy, collision-induced unfolding (CIU) plots can be constructed, which provide insights into the kinetic stability of the protein ion.^66–68^ This approach is conceptually analogous to probing stability by increasing temperature, which can be employed in solution to probe the melting-temperature of proteins at varying concentrations of urea.^69^ In the absence of solvent, the gas-phase stability of ions against CIU reflects the strength of intramolecular electrostatic interactions, including those between domains. It has been proposed that the number of CIU transitions correlates with the number of structural domains in a protein, allowing CIU to resolve sequential domain unfolding.^70^

In the absence of urea, activation of the +17 charge state of BSA results in a two-step unfolding process (CIU_50_ = 34.5 V and CIU_50_ = 47.5 V), producing extended gas-phase conformations (Fig. 3H and S10A). Upon addition of 1 M urea, under conditions where the intermediate structure is formed, a destabilization of the initial fold is observed. The first unfolding transition occurs at lower activation energies (CIU_50_ = 24.7 V) and leads to the formation of intermediate gas-phase structures not detected in the absence of urea (Fig. 3I and S10B). Higher amounts of activation lead to formation of extended structures with arrival times identical to those observed without urea. These results support the hypothesis that low concentrations of urea perturb interdomain contacts, lowering the energetic barrier for the initial unfolding transition.

### Modulating multi-step unfolding by ligand binding

The ability to distinguish different forms of a protein in the mass spectrum also opens the possibility to simultaneously follow the unfolding of proteins with and without a non-covalent ligand. This, in turn, allows for direct detection of how ligand binding influences protein stability and shift unfolding pathways. To explore this, we applied our method to human carbonic anhydrase I (CA), a protein that tightly coordinates a catalytically important Zn^2+^ ion in its active site. As the detection of low-molecular-weight ligand binding to proteins benefits greatly from increased MS resolution, we here employed a Q Exactive high-resolution mass spectrometer to demonstrate these capabilities.

**Fig. 4:**
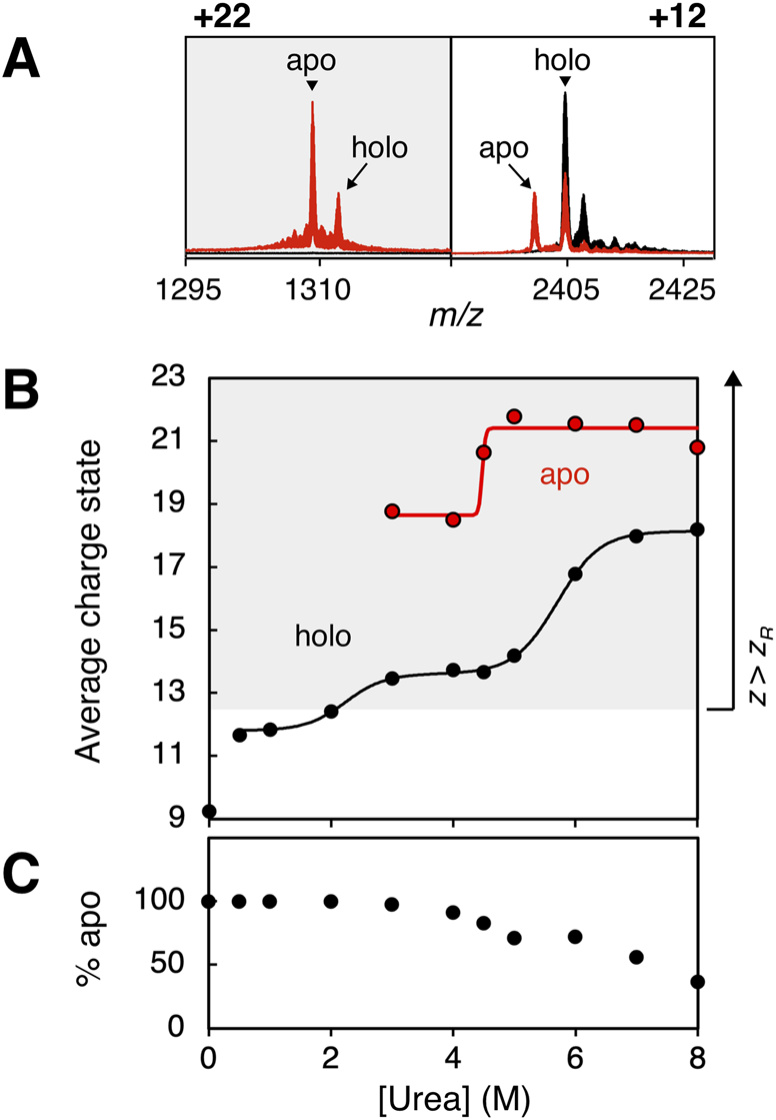
Monitoring ligand dissociation and unfolding of carbonic anhydrase I (CA). (A) The Zn^2+^-bound holo state and the Zn^2+^-free apo state are clearly detectable in the mass spectrum of CA. At 0 M urea (black) the protein populates lower charge states, and CA is detected only in its holo state. At 8 M urea (red) the CSD is shifted towards higher charge states and the apo state emerges, especially at higher charge states. (B) Unfolding curves for holo CA (black) and apo CA (red) constructed from fitting the CSD of the two protein forms. Charge states higher than the Rayleigh charge for CA (thus representing extended unfolded states) are marked in grey. The data points are fitted to sigmoidals. Note that the first unfolding transition for holo CA is not fitted, as not enough data points are available. (C) Relative population of the apo state. The apo state starts to become populated after the second unfolding transition of the holo form.

In the mass spectrum, the holo and apo forms of CA are clearly distinguishable, with the apo state emerging upon urea addition, particularly for higher charge states (Fig. 4A). We fitted the CSDs of the holo and apo forms separately to generate unfolding curves for each form (Fig. 4B). In the absence of urea, CA is detected exclusively in its fully zinc-bound holo state, exhibiting an average charge of +9.2 (0.73 *z*_R_). Urea addition induces formation of a fully Zn-bound intermediate with an average charge of +11.8 (0.94 *z*_R_), which in turn undergoes an additional conformational transition at a midpoint of 2.2 M urea (Fig. 4B, black). This transition is followed by a marked increase in the population of the zinc-free apo form (Fig. 4C), which also displays a significantly higher average charge state of + 18.6 (1.48 *z*_R_) compared to the holo state. This behaviour differs notably from that of Mb, where the aMb form appears at the onset of unfolding. In contrast, the apo form of CA only emerges following two distinct unfolding transitions. This likely reflects the buried nature of the Zn^2+^ binding site in CA, requiring partial unfolding for metal ion dissociation to occur.

Following the second unfolding transition, the apo and holo forms of CA are present at approximately equal abundance (Fig. 4C) but exhibit differing stability profiles. The apo form continues to unfold, with a transition midpoint at 4.5 M urea, reaching a highly extended conformation with an average charge state of + 21.4 (1.7 *z*_R_). In contrast, the holo form shows greater resistance to further unfolding, transitioning at 5.7 M urea to a more extended state (average charge: +18.1, 1.4 *z*_R_).

These findings demonstrate that ESI-MS can directly track the sequence of ligand dissociation and protein unfolding, revealing how ligand binding or release modulates the unfolding landscape of proteins. Such detailed, state-resolved information about ligand dissociation and conformational transitions would be challenging to obtain from in-solution spectroscopy experiments.

### Tracking multimer unfolding and disassembly

IMS-MS enables urea-induced unfolding experiments of complex protein systems where multiple assembly states coexist alongside significant conformational heterogeneity. One such system is the hormonal peptide insulin, which is active in its monomeric form but is stored in a hexameric state in the presence of Zn^2+^.^71^ Depending on solution state conditions, insulin is also known to form nonspecific oligomers, and to aggregate into large amyloid fibrils.^72,73^

**Fig. 5:**
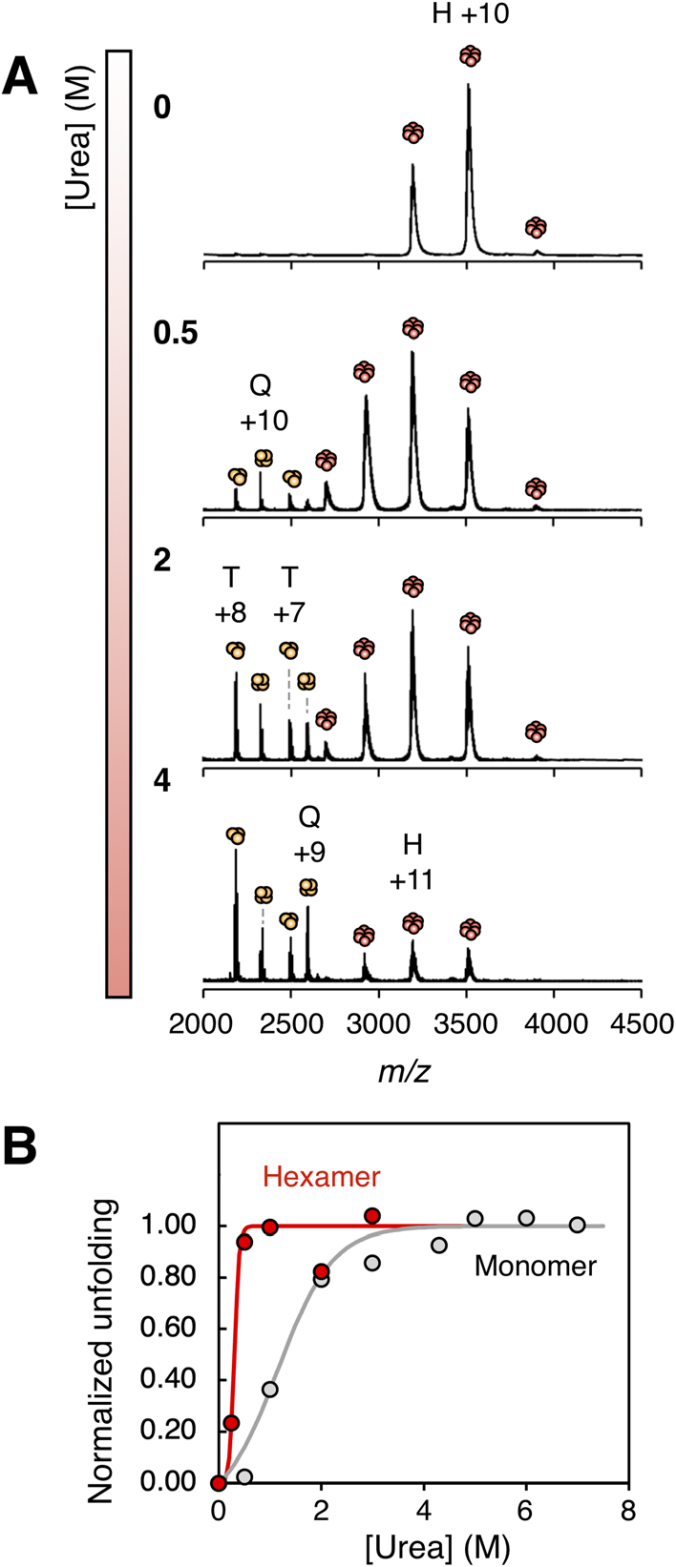
(A) nESI-MS of Zn^2+^-loaded insulin in 200 mM ammonium acetate (pH 6.8) in increasing amounts of urea. H = hexamer, Q = tetramer, T = trimer. (B) Normalized stability curves from monitoring the average charge state in gas-phase experiments of insulin hexamer (red circles) and monomers (grey circles). Data is fitted to sigmoidal functions (lines).

When analysed by nESI-MS under Zn^2+^-loaded conditions, insulin is detected as a mixture of monomers (M) and hexamers (H). Titration of urea into the Zn-bound insulin sample results in a slight CSD shift of the hexamer toward higher charge states, which indicates partial unfolding (Fig. 5A). This conformational change coincides with the appearance of smaller oligomeric species, including trimers (T) and tetramers (Q) (Fig. 5A, orange). Pentameric species are completely absent, which suggests that the observed subcomplexes are of solution-state origin rather than products of gas-phase dissociation. Collision induced dissociation of a destabilized insulin hexamer would be expected to produce primarily pentamers and monomers.^74^ We can also assume from this lack of pentamers in the mass spectrum that urea-induced dissociation of insulin proceeds *via* specific, non-random disassembly pathways in solution. As urea concentration increases, the relative abundance of the smaller oligomers rises as the hexamer signal intensity decreases. Additionally, the relative abundance of Zn^2+^-adducted trimers and tetramers increases with rising urea concentration (Fig. S11), indicating that Zn^2+^-bound insulin multimers are more stable than their metal-free counterparts.

By fitting the CSDs of the hexamer and monomer populations, unfolding curves for both assembly states can be extracted from the same dataset (Fig. 5B). These curves reveal that the hexamer undergoes an initial, steep cooperative unfolding transition with a midpoint at 0.3 M urea, reaching an average charge state of +11 (0.80 *z*_R_). This is followed by a much more gradual unfolding of the monomer with a transition midpoint at 1.1 M urea, resulting in an average monomer charge of 4.1 (0.73 *z*_R_) at the plateau phase. Notably, neither assembly state reaches charge states near *z*_R_, suggesting that even the unfolded forms remain relatively compact. This compactness is likely maintained by the presence of three intramolecular disulfide bonds in each insulin monomer, which greatly restrict chain expansion during denaturation.

**Fig. 6:**
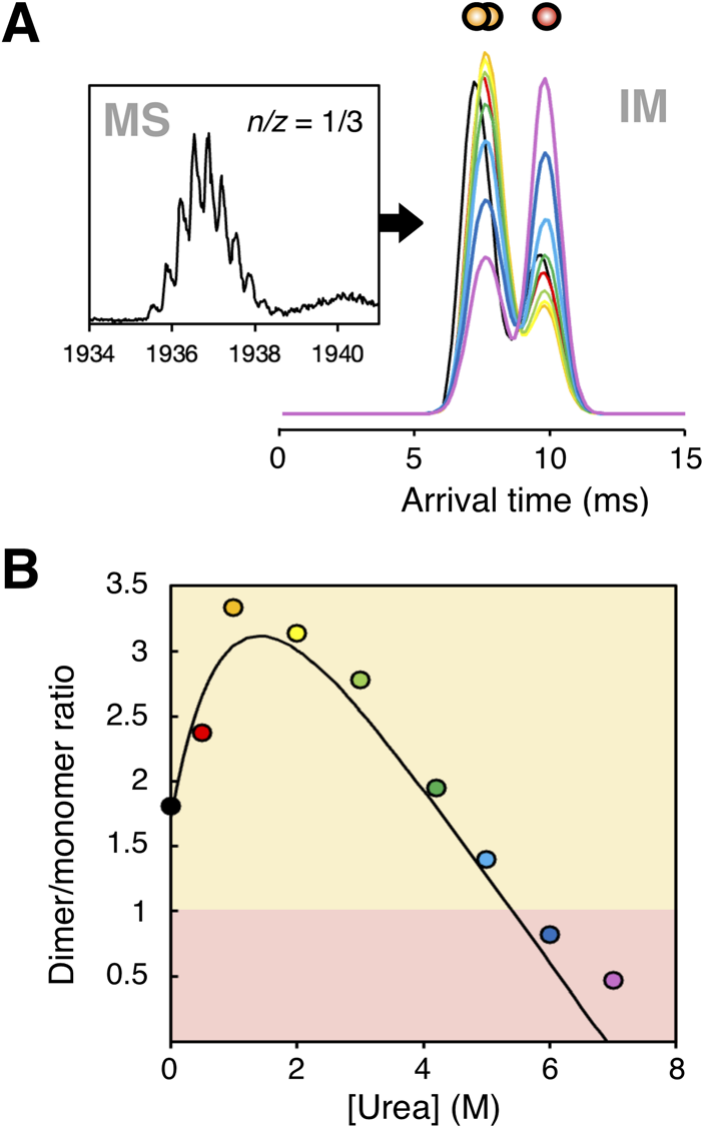
The +3 monomer and +6 dimer overlap in the *n*/*z* = 1/3 signal in the mass spectrum but can be resolved in the arrival time distribution of the ion mobility dimension. (A) Arrival time distribution for *n*/*z* = 1/3 at increasing urea concentrations. (B) Dimer/monomer ratio of the arrival time distribution in panel A. Scatter point colours correspond to the same colours in panel A. Data is fitted to a double exponential function (black line).

Interpretation of oligomeric species in mass spectra can be complicated by signal overlap in the *m*/*z* dimension. For example, an oligomer *n* with charge *z* can appear at the same *m*/*z* as an oligomer *2n* with charge 2*z*. IMS offers a solution by providing orthogonal separation of ions based on their CCS. In the case of insulin, the +3 monomer and the +6 dimer overlap in *m*/*z* but are clearly resolved in the IMS dimension (Fig. 6A). As urea concentration increases, the relative intensities of the monomer and dimer peaks shift in the arrival time distributions (Fig. 6B). In the absence of urea, the dimeric peak is approximately twice as intense as the monomeric peak. Upon urea titration, dimer abundance increases, peaking around 1 M urea, coinciding with the complete unfolding of the hexamer. Beyond this point, dimer abundance decreases as the monomer population rises, eventually becoming the dominant species as the monomer unfolds fully above 5 M urea.

These findings suggest a stepwise dissociation pathway for insulin in the presence of urea: the structured hexamer undergoes a conformational change and dissociates into smaller oligomers, which then further dissociate into compact, partially unfolded monomers at high urea concentration. Such detailed tracking of successive dissociation and unfolding events is not possible with in-solution spectroscopy techniques, which inherently average over coexisting species.

It has been found that the rate of insulin aggregation drastically increased between 0 and 2 M urea due to an increased population of partially unfolded monomers.^75^ Excellent agreement is found between our data on hexamer dissociation, monomer unfolding, and these literature values on insulin aggregation rates (Fig. S12). Together, these results demonstrate that native IMS-MS, when combined with controlled chemical denaturation, provides a powerful platform for dissecting protein structural rearrangements. This approach not only captures intermediate states that are invisible to ensemble-averaging techniques but can also link structural dynamics to functional outcomes such as protein aggregation.

## Conclusions

We demonstrate that urea-induced unfolding, a well-established method in biochemistry for studying protein stability, can be effectively and readily coupled with ESI-MS. This work adds to the growing suite of adaptations that extend ESI-MS to structural studies under more standard biochemical conditions. Other developments in this area include the use of non-volatile buffers,^45,47^ direct analysis of proteins from unpurified cell lysates,^76,77^ and temperature induced unfolding experiments in solution.^30,54,78^ However, while thermal denaturation provides a straightforward way to induce unfolding, it is often limited by rapid protein aggregation above the melting temperature.^80^ In contrast, urea-induced denaturation is frequently more reversible and avoids this limitation, allowing unfolding to be accurately monitored even for proteins prone to aggregate at high temperature, such as BSA, or in some solution environments, such as insulin.

We show that the charge state distribution serves as a sensitive reporter of urea-dependent changes in protein conformation in solution. These changes can be reliably tracked on a Synapt QTOF, an Orbitrap Q Exactive, and in principle any mass spectrometer equipped with a nESI source. While ion mobility is not required to follow conformational changes upon unfolding, it does provide orthogonal separation of overlapping signals of proteins with multiple assembly states and enables probing of the kinetic stability of urea-destabilized states through CIU experiments.

This approach further bridges the fields of analytical chemistry and life science by using standard sample conditions commonly employed in traditional biochemistry and biophysics, offering a valuable tool for ensemble-resolved studies of protein folding and unfolding. It provides structural insights into both conformational and assembly intermediates at low micromolar protein concentrations and does not require high sample purity, fluorescent amino acids, or any type of protein labeling. Additionally, the sample volume needed for a single data point in an unfolding curve is 10–50 times smaller than what is needed for condensed-phase spectroscopy.

We also find that the quality of the nESI emitters is critical for obtaining high-quality mass spectra at high urea concentrations. The emitters used in this work are approximately ten times smaller than those used in earlier studies combining urea with ESI-MS, though not as small as the emitters typically employed for ionization from non-volatile salt solutions. Such submicron emitters could potentially improve performance further and could possibly extend the method to less volatile osmolytes such as guanidinium chloride. However, submicron emitters also pose practical challenges, including more difficult sample loading, increased chances of clogging, and reduced spray stability.

Taken together, these results highlight the flexibility of using nESI-MS to study protein stability in the presence of urea. By combining structural detail with low sample requirements and compatibility with standard biochemical conditions, and without requiring specialized electrospray sources, this method creates new possibilities for exploring folding pathways across a broad range of proteins, including those difficult to analyse with traditional techniques.

## Author contributions

Conceptualization: N. Ö. and J. S. J. Methodology: N. Ö. and J. S. J. Formal analysis: N. Ö., J. S. J. and G. P. S. Funding acquisition: N. Ö. and K. P. Investigation: N. Ö., J. S. J., E. R. and G. P. S. Project administration: K. P. Supervision: K. P. Validation: N. Ö., J. S. J., and E. R. Visualization: N. Ö. Writing – original draft: N. Ö. and J. S. J. Writing – review & editing: K. P., G. P. S. and E. R.

## Conflicts of interest

There are no conflicts to declare.

## Supplementary Material

SC-OLF-D5SC05773F-s001

## Data Availability

Data supporting this article have been included as part of the supplementary information (SI). Supplementary information is available. See DOI: https://doi.org/10.1039/d5sc05773f.
