# Supplementary material for: Mass spectrometry detects folding intermediates populated during urea-induced protein denaturation
Source: Chem Sci. 2025 Oct 23;16(47):22407–16. doi: 10.1039/d5sc05773f (PMC12573884; doi:10.1039/d5sc05773f)
Supplement: SC-016-D5SC05773F-s001 [file SC-016-D5SC05773F-s001.pdf]

Supplementary Information (SI) for Chemical Science.
This journal is © The Royal Society of Chemistry 2025

Supplementary information for

# Mass spectrometry detects folding intermediates populated during urea-induced protein denaturation

Nicklas Österlund, [a,b,†] Jacob S. Jordan,[a,b,†] Eleonora Renzi [a,b] , Gergo Peter Szekeres [a,b], and Kevin Pagel [a,b,*]

a. Institute of Chemistry and Biochemistry, Freie Universität Berlin, Berlin, Germany.
b. Department of Molecular Physics, Fritz Haber Institute of the Max Planck Society, Berlin, Germany.

***To whom correspondence should be directed:** Kevin.pagel@fu-berlin.de

**EXPERIMENTAL** **2**
Sample preparation 2
Mass spectrometry 2
Imaging of nESI emitters 3
Calculation of theoretical Rayleigh charge 3
Fluorescence spectroscopy 4
Circular dichroism spectroscopy 4
Fitting of unfolding curves 4

**SUPPLEMENTARY FIGURES** **5**
Fig S1. Ion mobility profiles of myoglobin during urea induced unfolding. 5
Fig S2. Mass spectrum of myoglobin on a Q Exactive UHMR instrument. 6
Fig S3. nESI spectra of myoglobin in non-volatile salt solutions. 7
Fig S4. Comparison of different nESI emitters. 8
Fig S5. Reproducibility of myoglobin unfolding from independent replicates. 9
Fig S6. Stability curve for myoglobin determined from charge state distributions. 10
Fig S7. Quantifying the amount of folded myoglobin. 11
Fig S8. Mass spectrometry to follow unfolding of cytochrome c at pH 3. 12
Fig S9. CD spectroscopy to follow BSA unfolding. 13
Fig S10. CIU plots of BSA with fitted CIU50 values. 14
Fig S11. Relative intensity of zinc-free and zinc-bound insulin oligomers. 15
Fig S12. Insulin hexamer abundance vs. insulin aggregation rate. 16

## Experimental

### Sample preparation

Myoglobin from equine skeletal muscle, cytochrome c from bovine heart, bovine serum albumin (BSA), and human carbonic anhydrase I were purchased from Sigma-Aldrich, dissolved in Milli-Q water, and stored at 4 °C until analysis. Human recombinant insulin was purchased from Sigma-Aldrich and initially dissolved in a dilute solution of acetic acid. The insulin solution was subsequently fractionated using a Superdex 75 10/300 size-exclusion chromatography column (Cytiva) equilibrated with 200 mM ammonium acetate at pH 6.8. Fractions corresponding to monomeric insulin were collected, flash-frozen in liquid nitrogen, and stored at –80 °C until use.

The concentrations of protein stock solutions were measured by UV-Vis spectroscopy at 280 nm using a DS-11 microvolume spectrophotometer (DeNovix). Extinction coefficients for the respective proteins were calculated from their amino acid sequences. Protein solutions were then further diluted to 100 μM and, in the case of insulin, loaded with 100 μM zinc acetate. Protein samples were mixed with stock solutions of ammonium acetate and urea just prior to measurements, obtaining samples of 15 μM protein, 200 mM ammonium acetate pH 6.8 and 0-8 M urea. Urea stock solutions were always prepared fresh.

### Mass spectrometry

Mass spectra and ion mobility arrival time distributions of myoglobin, cytochrome c, bovine serum albumin, and insulin were acquired in positive mode on a Synapt G2S quadrupole-time-of-flight (Q-TOF) mass spectrometer equipped with a travelling wave ion mobility (TWIMS) cell (Waters Corp., USA). A cone voltage of 20 V and a source offset of 5 V was used. The source temperature was kept at 25 °C, source temperature and the gas flow to the trap, helium, and IMS cells were kept at 5 ml/min, 180 ml/min, and 90 mL/min respectively. Trap voltage was kept at 4 V except in Collision-induced unfolding (CIU) experiments where it was increased stepwise. Mass spectra and ion mobility arrival time distributions were analyzed using MassLynx V4.2 (Waters Corp., USA). CIU data were analyzed and plotted using CIUSuite 2.[1]

TWIMS arrival times were calibrated to collision cross section values using the methods of Bush et al.[2] and Thalassinos et al.[3] Calibration curves were constructed using myoglobin, cytochrome c, and bovine serum albumin charge states sprayed from 200 mM ammonium acetate.

Mass spectra of carbonic anhydrase I was acquired on an Orbitrap Q Exactive UHMR (ThermoFisher Scientific, USA). Spectra were acquired in positive mode at a resolution of 100,000. Ion transmission and detector optimization were set to “low *m/z*” and a trapping gas pressure of 1.0 (arb. units) was used for sufficient transmission and desolvation of protein ions. The heated capillary was set to 250 °C. The source offset was set to 21 V and in source trapping was turned on with a desolvation voltage of -10 V. Mass spectra were analyzed using Freestyle V.1.8 (ThermoFisher Scientific, USA).

Samples were in both cases ionized using nano-ESI sources, from borosilicate emitters prepared in-house on a P-1000 micropipette puller (Sutter Instrument, USA). Ionization was initiated by applying a capillary voltage of 0.8-1.2 kV to a platinum wire inserted into the emitter and in contact with the sample solution.

Mass spectra were typically acquired for 1–5 minutes, yielding 60–300 independent mass spectra per sample replicate. The analyzed spectra represent the average of these measurements.

### Imaging of nESI emitters

Optical microscopy images were acquired in transmission mode by a home-built long working distance setup of ~25X magnification equipped with a CMOS camera. In bright-field images, the capillaries were illuminated with diffuse white light source at 180° from the objective, while in dark-field images, the diffuse light source was shifted to reach high contrast. The field of view was calibrated by imaging a 1 mm stage micrometer target featuring 10 μm divisions.

### Calculation of theoretical Rayleigh charge

The Rayleigh charge ($z_R$) is the maximum charge a droplet can carry before it undergoes fission. At the final stage of ESI, a protein is believed to acquire a charge which is lower or equal to $z_R$ of a droplet with the size of the protein itself. $z_R$ for a spherical droplet is given by **(I)**, where e is the elementary charge, $\varepsilon_0$ is the vacuum permeability, $\gamma$ is the surface tension of the droplet, and r is the radius of the droplet.

$$z_R = \frac{8\pi}{e}\sqrt{\epsilon_0 \gamma r^3} \qquad \textbf{(I)}$$

The radius of a sphere can be defined from the volume (V) of the sphere, which in turn depends on the mass and density (ρ) of the material **(II)**. For molecules, molar mass (M) is a convenient measure of mass. This conversion introduces Avogadros constant ($N_A$) in the equation.

$$r^3 = \frac{3V}{4\pi} = \frac{3M}{4\pi\rho N_A} \qquad \textbf{(II)}$$

Inserting **(II)** into **(I)** yields **(III)**

$$z_R = \frac{8\pi}{e}\sqrt{\frac{\epsilon_0 \gamma 3M}{4\pi\rho N_A}} \qquad \textbf{(III)}$$

For proteins we here assume ρ = 1.3 g/cm$^3$, which has been shown to be a reasonable assumption,[4,5] and a surface tension of 0.072 N/m, corresponding to the surface tension of water at 298 K. This yields a numerical relationship **(IV)** between the molecular weight of a protein (in Da) and its Rayleigh charge.

$$z_R \approx 0.074\sqrt{M} \qquad \textbf{(IV)}$$

This relationship was used to calculate $z_R$ for myoglobin (+9.8), cytochrome c (+8.3), bovine serum albumin (+19), carbonic anhydrase (+12.6), the insulin monomer (+5.6), and the insulin hexamer (+13.8).

### Fluorescence spectroscopy

Intrinsic tryptophane fluorescence was measuring under identical sample conditions as MS experiments (15 µM protein, 200 mM ammonium acetate pH 6.8, with varying amounts of urea). Fluorescence measurements were performed in triplicate using 100 µL samples loaded into a Corning 96-well non-binding flat-bottom half-area microplate. Measurements were acquired with a Spark multimode plate reader (Tecan, Switzerland), using excitation at 280 nm. Emission was recorded from the top of the plate at 338 nm and 354 nm.

### Circular dichroism spectroscopy

Circular dichroism (CD) in the far-UV range was measured for bovine serum albumin upon addition of urea using a DSM 20 spectrometer (On-Line Instrument Systems, Inc., USA). 15 µM protein in 200 mM ammonium acetate pH 6.8 was measured from a 1 mm quartz cuvette. The ellipticity between 260 and 200 nm (1 nm step size, 4 s sampling time per point) was recorded for each urea concentration. Only data points with a PMT voltage < 600 were considered.

### Fitting of unfolding curves

Stability curves were generated from the emission intensities and fitted to 4-parameter logistic sigmoid fits (**V**) using a custom Python script.

$$y = y_0 + \frac{L - y_0}{1 + e^{-k(x - x_0)}} \quad \textbf{(V)}$$

Where $y_0$ is the baseline, L is the upper asymptote, $x_0$ is the inflection point, and k is the slope.

For proteins that display two unfolding steps, a multi-component sigmoidal was fitted according to **(VI)**.

$$y = y_1 + y_2 \quad \textbf{(VI)}$$

Where $y_1$ and $y_2$ are 4-parameter logistic sigmoid fits according to **(V)**.

## Supplementary Figures

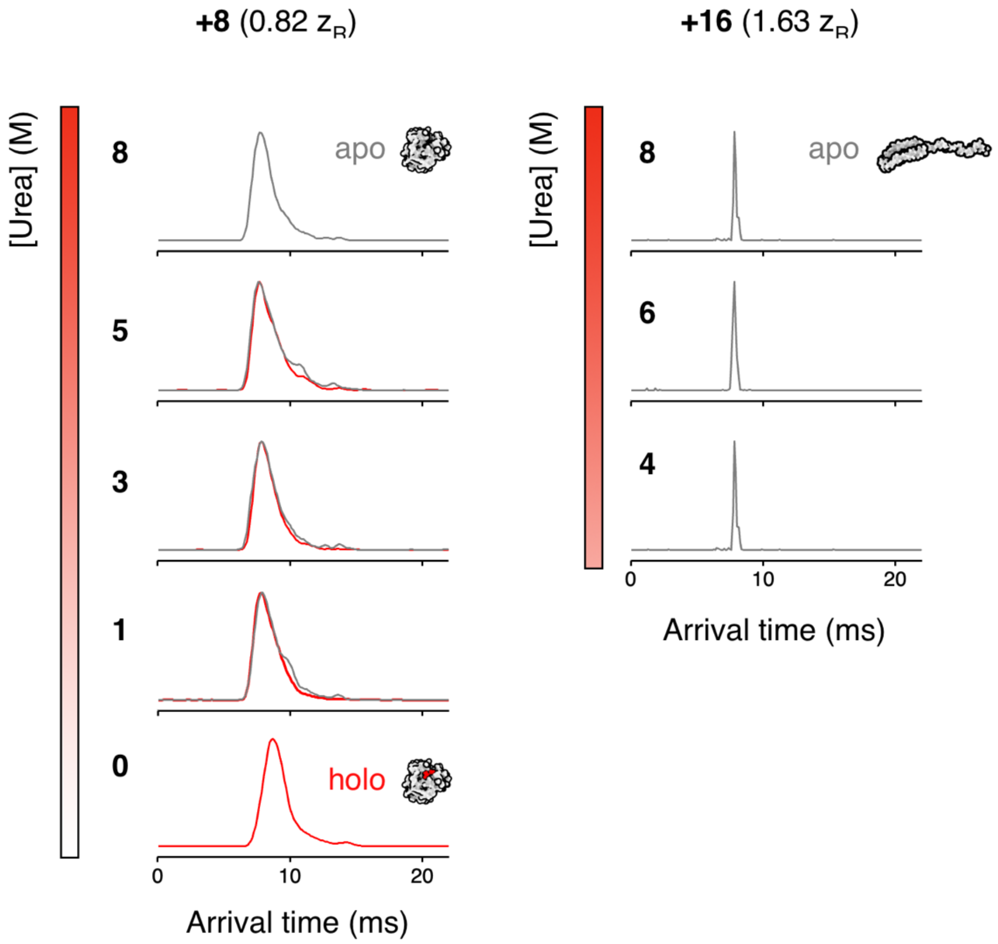


**Fig S1. Ion mobility profiles of myoglobin during urea induced unfolding.**

Arrival time distributions of the most intense compact native-like (left) and extended unfolded (right) charge state for myoglobin as a function of urea concentration. The signal from the holo form is shown in red and the signal from the apo form is shown in gray. Only minor shifts in the arrival time distributions are seen as the urea concentration is increased, both for the native-like and the unfolded state.

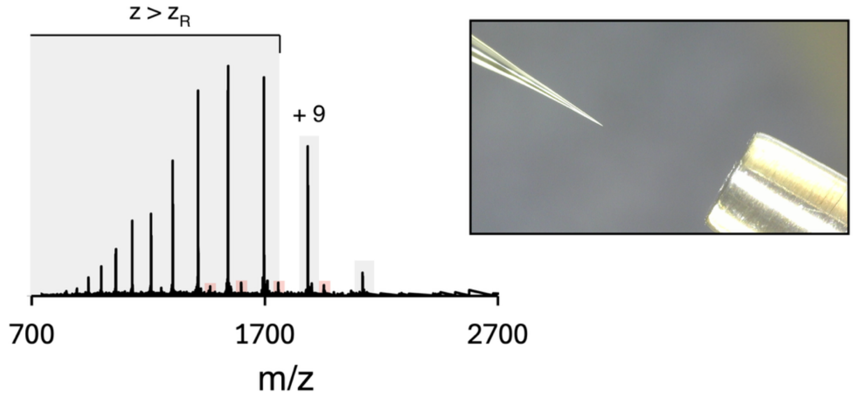


**Fig S2. Mass spectrum of myoglobin on a Q Exactive UHMR instrument.**

Mass spectrum of Mb in 8 M urea analyzed on an orbitrap system. Heme-bound myoglobin (hMb) is highlighted in red, heme-free (aMb) myoglobin is highlighted in grey. Signals where the ESI charge (z) is higher than the theoretical Rayleigh charge ($z_R$) for myoglobin (+9.8) are indicated. The amount of detected unfolding is similar to what was detected on the Waters Synapt system (Fig 1). The insert shows the orientation of the nESI needle in relation to the MS inlet, which is on-axis in contrast to the Waters source where the needle is oriented at 90° relative to the inlet.

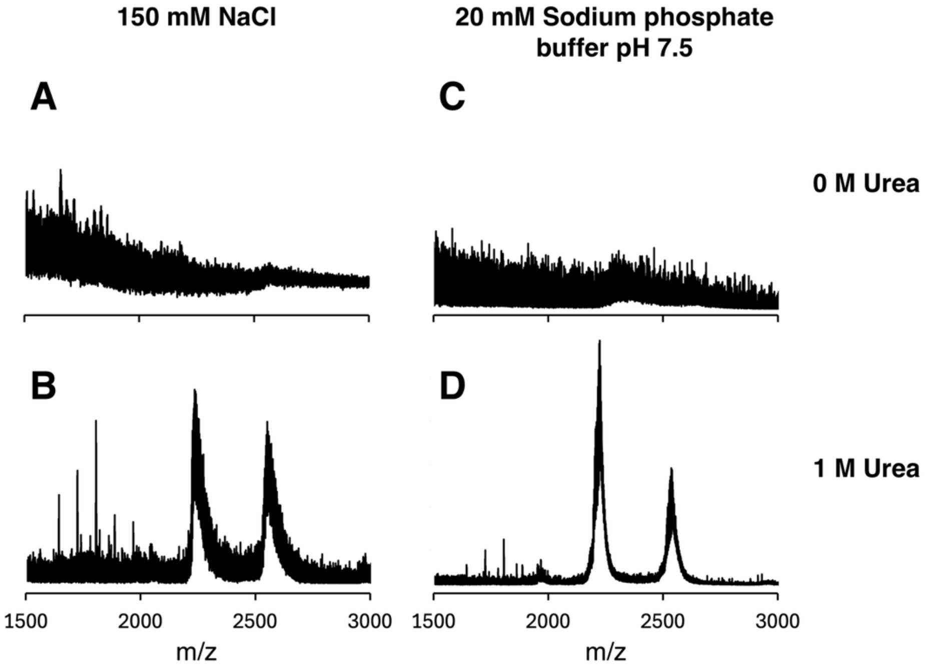


**Fig S3. nESI spectra of myoglobin in non-volatile salt solutions**.

Mass spectra of myoglobin in 150 mM NaCl (A,B), and in 20 mM sodium phosphate buffer pH 7.5 (C,D). Either without (A,C) or with (B,D) urea. In the presence of urea, similar charge state distributions are detected as is detected in 200 mM ammonium acetate pH 6.8, but the detected peaks are considerably more salt adducted.

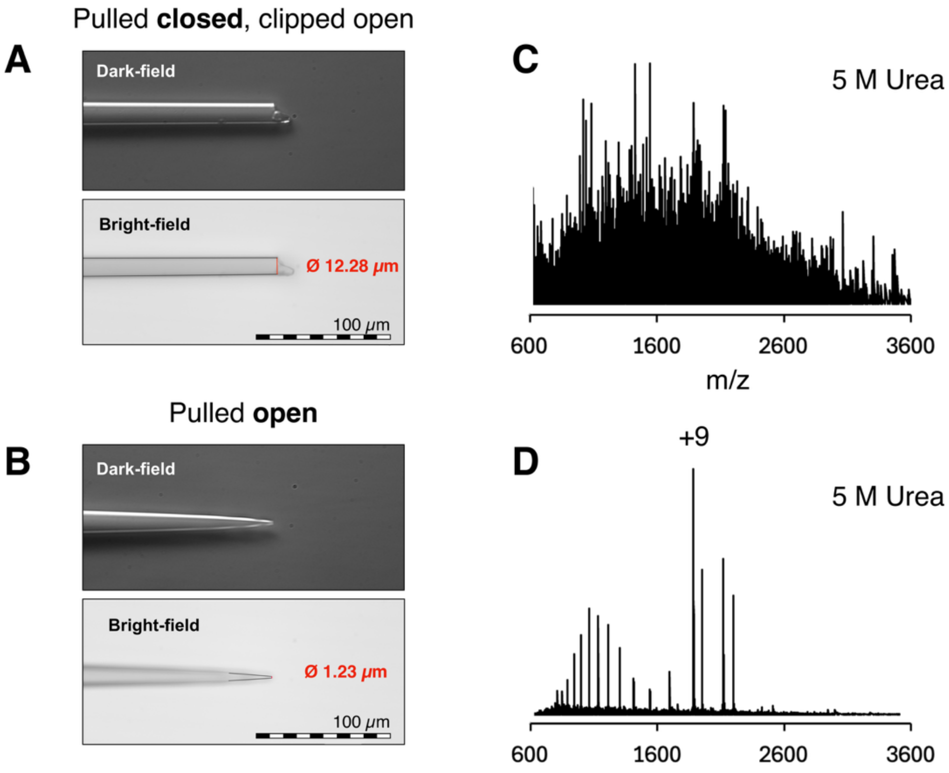


**Fig S4. Comparison of different nESI emitters.**

(A,B): Light microscopy images of borosilicate nESI emitters pulled either closed and then manually clipped open (A) or open (B). Measured diameter is shown in red, with open needles having approximately 10 times smaller emitter openings, and a much smoother emitter opening. (C,D): Mass spectra of myglobin in 200 mM ammonium acetate pH 6.8 with 5 M urea sprayed from needles pulled closed and then manually clipped open (A) or open (B). Only the emitters that were manufactured with open tips were able to produce well resolved mass spectra under these conditions.

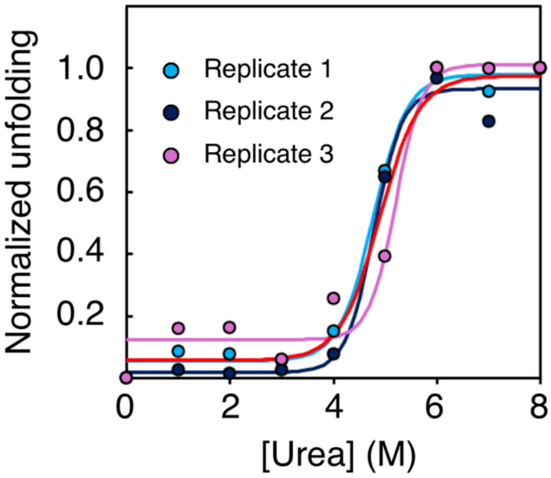


| | 1 | 2 | 3 | CV |
|---|---|---|---|---|
| **Midpoint** | 4.8 M | 4.8 M | 5.2 M | 5.1 % |
| **Steepness** | 3.0 $M^{-1}$ | 3.7 $M^{-1}$ | 3.8 $M^{-1}$ | 12.7 % |

**Fig S5. Reproducibility of myoglobin unfolding from independent replicates.**

Three independent replicates of myoglobin unfolding (followed by monitoring the apo/halo ratio by ESI-MS) plotted and fitted individually. The obtained values for the unfolding midpoint and the steepness of the transition are reported, together with the coefficient of variation (CV) for the fitted parameter. CV is calculated as standard deviation of the replicates divided by the mean of the replicates times 100.

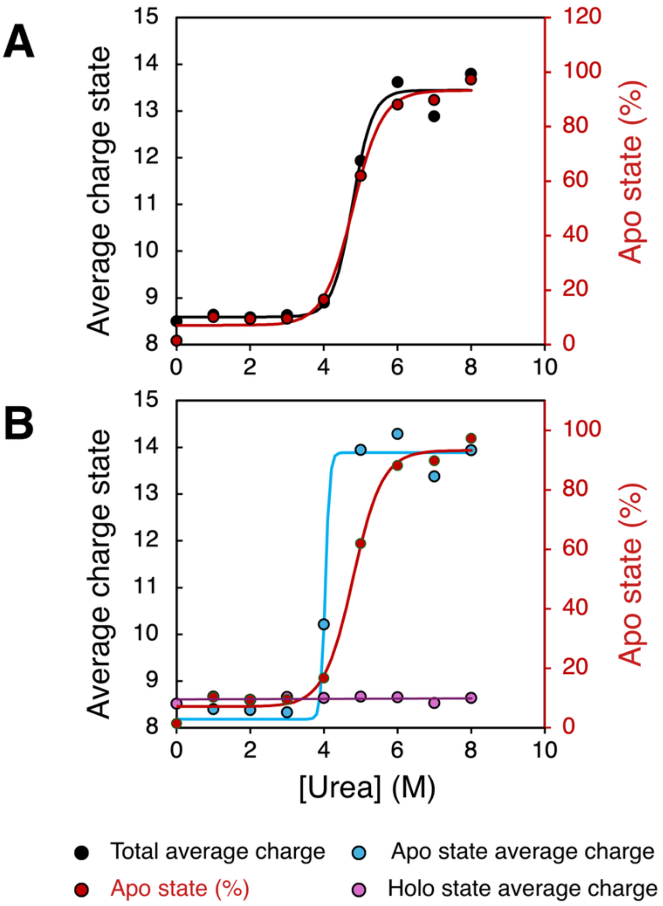


**Fig S6. Stability curve for myoglobin determined from charge state distributions.**

A) Comparison between the unfolding curve constructed from tracking the holo/apo ratio of myoglobin (red) and the unfolding curve constructed from the total average charge state (of both apo and holo forms of myoglobin). B) Comparison between the unfolding curve constructed from tracking the holo/apo ratio of myoglobin (red) and the unfolding curves constructed from the average charge of the apo state (blue) and the holo state (purple).

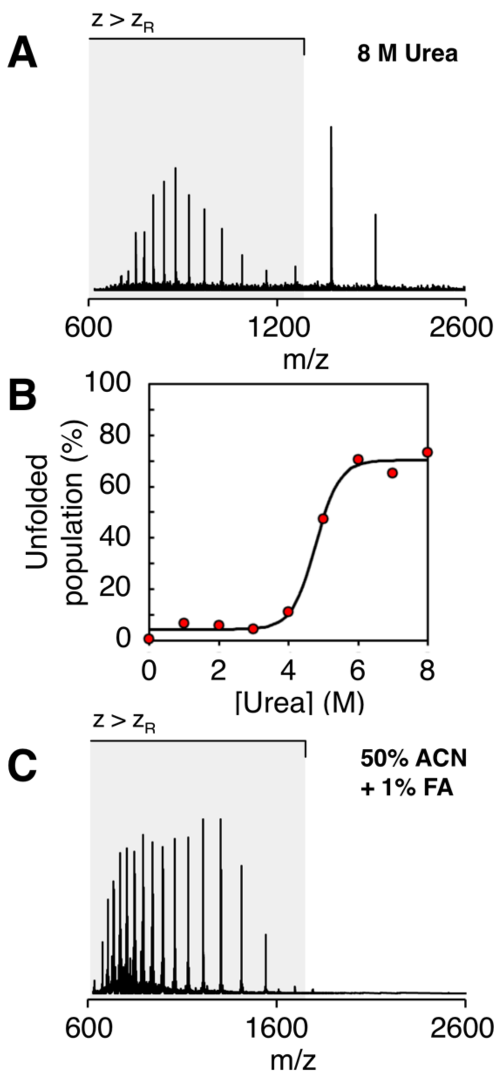


**Fig S7. Quantifying the amount of folded myoglobin.**

A) Mass spectrum of myoglobin in 200 mM ammonium acetate pH 6.8 with 8 M urea. Charge states with a higher charge than the theoretical Rayleigh charge for myoglobin (+ 9.8) are highlighted in grey. This shows that a sub-population of myoglobin remains in a compact state even at 8 M urea. B) Unfolding plot constructed by dividing the intensity sum of all peaks above $z_R$ (panel A, grey box), by the intensity sum of all peaks. This shows that approximately 70% percent of myoglobin is unfolded at the unfolding plateau, while 30% remains in a compact conformation. C) Mass spectrum of myoglobin under typical denaturing MS conditions (50% acetonitrile, 1% formic acid), showing that the protein ensemble is fully shifted towards unfolded states with $z > z_R$, which is in contrast to the observation for urea induced unfolding.

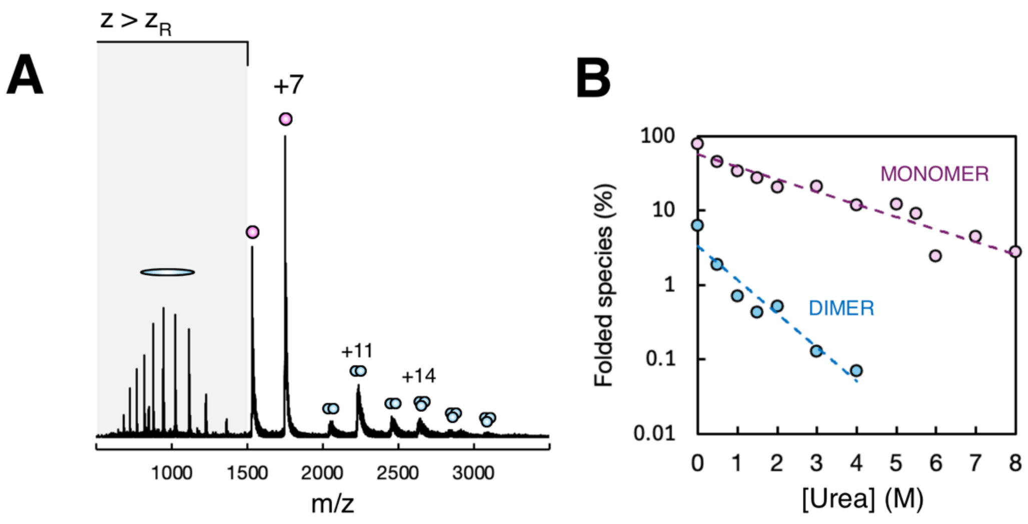


**Fig S8. Mass spectrometry to follow unfolding of cytochrome c at pH 3.**

A) Mass spectrum of cytochrome c in 200 mM ammonium acetate, without urea, adjusted to pH 3 with acetic acid. Compact monomers (pink spheres) extended monomers (blue rod), as well as oligomers (blue spheres) are detected. B) Unfolding of monomers and dimers can be followed individually in the same experiment. The amount of folded monomer and dimer (total signal with $z < z_R$) is followed as the concentration of urea is increased. The dimer is less stable than the monomer, and no dimer is detected above 4 M urea.

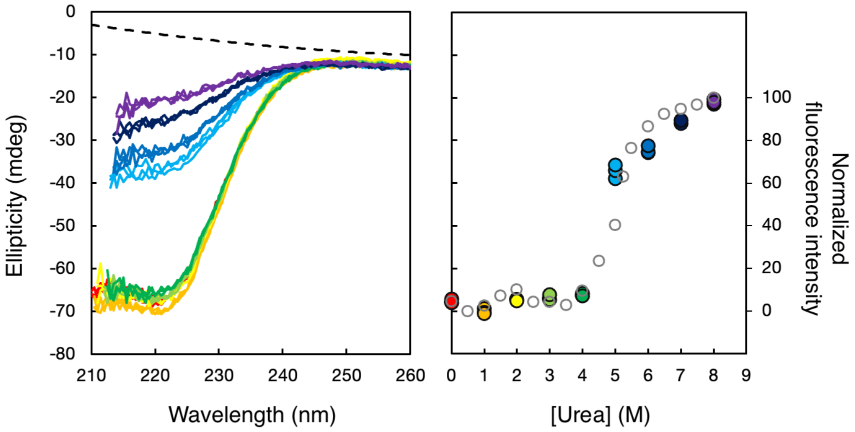


**Fig S9. CD spectroscopy to follow BSA unfolding.**

CD spectrometry of 15 µM bovine serum albumin in 200 mM ammonium acetate pH 6.8 upon increasing the urea concentration from 0 to 8 M. The raw CD spectra are shown on the left, data points with a PMT voltage > 600 are excluded. The blank spectrum is shown as a dashed black line. The ellipticity at 222 nm is plotted on the right as a function of urea concentration, a sharp unfolding transition is seen above 4 M urea, this agrees well with fluorescence data (open circles).

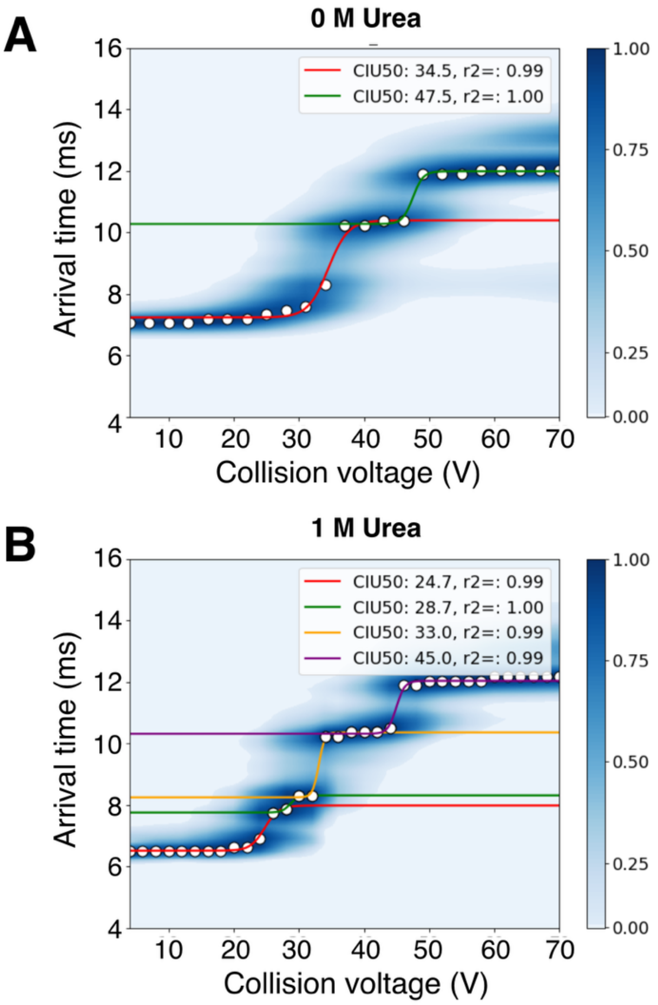


**Fig S10. CIU plots of BSA with fitted CIU50 values.**

CIU plots of bovine serum albumin +17 charge state (also shown in Fig 3) fitted in CIUSuite 2 to obtain CIU50 values for the unfolding transitions. A) In 0 M urea BSA undergoes two transitions, at trap voltage 35 and 48 V. B) In 1 M the destabilized unfolding intermediate undergoes two additional unfolding transitions at lower trap voltage, at 25 and 29 V, in addition to transitions that occur at similar trap voltage as in A.

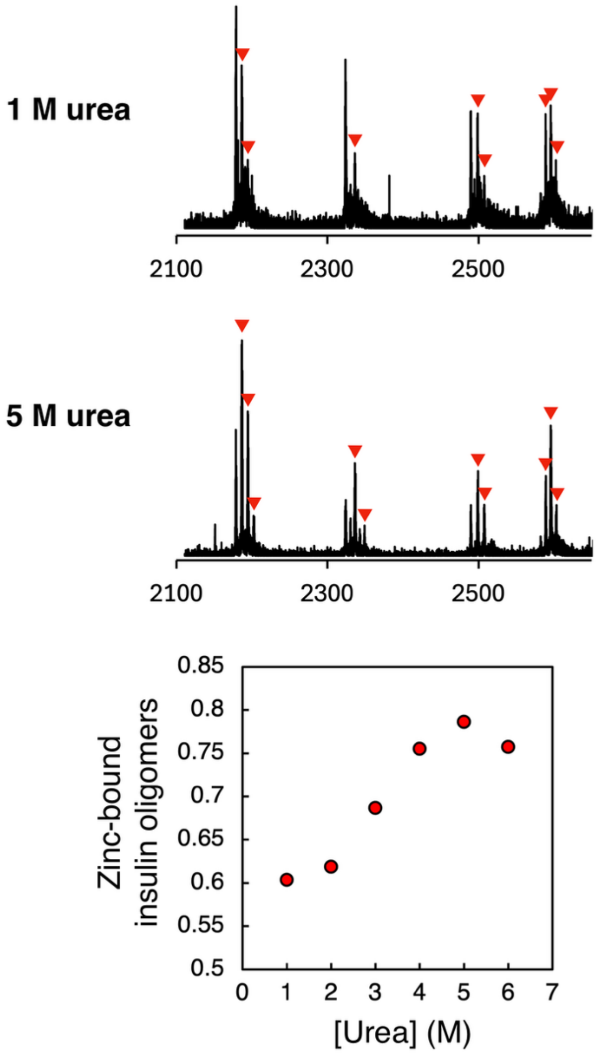


**Fig S11. Relative intensity of zinc-free and zinc-bound insulin oligomers.**

The region of the insulin mass spectrum where trimeric and tetrameric insulin oligomers are detected. Zinc-adducted oligomers are marked with red triangles. The relative abundance of zinc-adducted forms increases between 2 and 5 M urea and then decreases slightly.

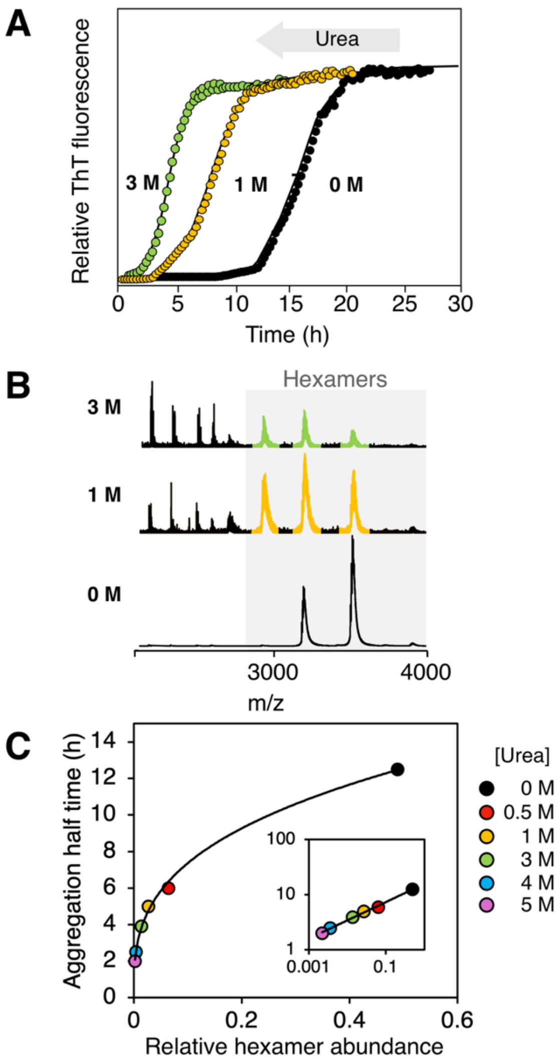


**Fig S12. Insulin hexamer abundance vs. insulin aggregation rate.**

Comparing the detected dissociation of insulin hexamers to literature data on insulin aggregation in urea. A) Thioflavin T aggregation assay follows the amyloid aggregation process when urea is added to human insulin in increasing concentration. Data retrieved from Ahmad et al,[6] reproduced under the Creative Commons license. B) Tracking hexamer dissociation in this work, increasing the urea concentration leads to dissociation of insulin hexamers. C) Dissociation of insulin hexamers (this work) is correlated to an increase in aggregation rate (literature value)[6]. A log-log representation of the same plot is shown as an insert.

## References


1 D. A. Polasky, S. M. Dixit, S. M. Fantin and B. T. Ruotolo, *Anal Chem*, 2019, **91**, 3147–3155.

2 M. F. Bush, Z. Hall, K. Giles, J. Hoyes, C. V. Robinson and B. T. Ruotolo, *Anal Chem*, 2010, **82**, 9557–9565.

3 T. Hunter, T. Pawson, J. D. Scott, G. F. Audette, R. Engelmann, W. Hengstenberg, J. Deutscher, K. Hayakawa, J. W. Quail, L. T. Delbaere, O. N. Jensen, F. Kjeldsen, M. M. Savitski, M. L. Nielsen, L. Shi, R. A. Zubarev, R. Aebersold, M. Mann, S. B. ; J. Ficarro, J. Villen, S. A. Beausoleil, S. A. Gerber, S. P. Gygi, A. Chi, C. Huttenhower, L. Y. Geer, J. J. Coon, J. E. Syka, D. L. Bai, J. Shabanowitz, D. J. Burke, O. G. Troyanskaya, D. F. Hunt, H. Molina, D. M. Horn, N. Tang, S. Mathivanan and A. Pandey, *Anal Chem*, 2008, **81**, 248–254.

4 K. C. B. De Freitas, *J Am Soc Mass Spectrom*, 2018, **29**, 2059–2066.

5 H. P. Erickson, *Biol Proced Online*, 2009, **11**, 32–51.

6 A. Ahmad, I. S. Millett, S. Doniach, V. N. Uversky and A. L. Fink, *Journal of Biological Chemistry*, 2004, **279**, 14999–15013.
